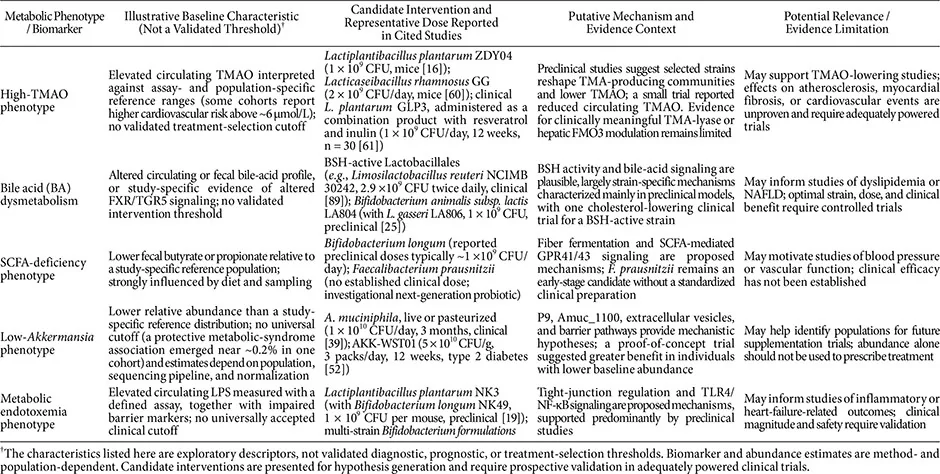# Probiotics in Cardiometabolic Diseases: Current Evidence, Postbiotic Perspectives, and Future Directions

**DOI:** 10.4014/jmb.2605.05001

**Published:** 2026-09-02

**Authors:** Fan Yang, Wanting Qin, Guoxia Zhang, Xinye Li, Xinyu Yang, Li Zhou, Yanwei Xing

**Affiliations:** 1 Hospital of Chengdu University of Traditional Chinese Medicine, Chengdu, P. R. China; 2 Dongzhimen Hospital Affiliated to Beijing University of Chinese Medicine, Beijing 100700, P. R. China; 3 Institute of Acupuncture and Moxibustion, China Academy of Chinese Medical Sciences, Beijing 100700, P. R. China; 4 Dongfang Hospital Affiliated to Beijing University of Chinese Medicine, Beijing 100700, P. R. China; 5 Guang’anmen Hospital, Chinese Academy of Chinese Medical Sciences, Beijing 100053, P. R. China

**Keywords:** Cardiometabolic diseases, Probiotic, Postbiotics, Gut microbiome therapy, *Akkermansia muciniphila*, Precision medicine

## Abstract

The rise in urbanization and sedentary lifestyles, along with the aging of the global population, is contributing to an increasing burden of life-threatening diseases. The cardiometabolic disease (CMD) spectrum, which includes heart failure, hypertension, coronary heart disease, and diabetes, is closely associated with metabolic syndrome components such as obesity and elevated hepatic lipid accumulation. The gut microbiome mediates these disorders through multiple signaling axes. Imbalance of the intestinal microbiota is closely linked to the pathogenesis of atherosclerosis and hypertensive vascular remodeling. Supplementation with probiotics — and, more recently, their derived postbiotic products — can alter the composition and functional metabolic outputs of the gut ecosystem, offering a mechanistically informed approach for CMD management. This review delineates the mechanisms of action of six well-studied probiotics (*Akkermansia muciniphila*, *Bifidobacterium*, *Lactobacillus*, *Bacillus*, *Enterococcus*, and *Lactococcus lactis*), with selected discussion of their postbiotic derivatives where evidence is available. We further examine how antimicrobial and metabolic interventions integrated through the gut-heart-liver axis may improve clinical outcomes and attenuate disease progression. The advancement of precision cardiometabolic medicine through the combined effects of these therapies may facilitate the development of personalized treatment strategies.

## Introduction

Due to rapid urbanization, the increasing prevalence of unhealthy lifestyles, and population aging, cardiometabolic diseases (CMD) have become a major global health concern [1]. The CMD spectrum includes heart failure, hypertension (HTN), coronary heart disease, and diabetes. These conditions arise from a variety of interrelated metabolic disturbances, including obesity, non-alcoholic fatty liver disease (NAFLD), disorders of amino acid metabolism, thyroid dysfunction, and alterations in gut microbiota-derived metabolites [2, 3]. The prevalence of CMDs until the year 2022 can be described as one of the biggest public health concerns at this time. In fact, almost half of the deaths that take place due to non-communicable diseases occur due to this reason. Likewise, almost a quarter of the total disability-adjusted life years or DALY lost during the year 2019 have been due to CMDs [4]. The growing burden of cardiometabolic disease has catalyzed the emergence of Cardiometabolic Medicine as a rapidly evolving interdisciplinary subspecialty that is reshaping cardiovascular care [5]. Ongoing research aimed at better understanding CMDs, improving risk stratification, and developing effective therapeutic strategies continues to yield novel intervention approaches with clinically proven benefits that challenge established paradigms [6].

In parallel, microbiome research has established the gut microbiota as a key regulator of organism-wide metabolism and immunity. Imbalance of intestinal microecology is associated with cardiovascular diseases (CVDs), particularly atherosclerosis (AS), hypertensive vascular remodeling, and endothelial dysfunction, through the gut-heart-liver axis [7]. Pathological dysbiosis damages intestine barrier function, increases permeability, and allows translocation of microbial products (*e.g.*, lipopolysaccharide) into circulation. This results in chronic low-grade inflammation and metabolic endotoxemia and thus accelerates the progression of various CVDs [8]. In this way, the targeted modulation of the gut ecosystem represents a promising avenue for CMD therapeutics. The addition of probiotic and postbiotic supplementation, through the modulation of a microbial composition as well as functional metabolic outputs, represents a mechanistically informed approach to restoring homeostasis and diminishing disease trajectories [7].

According to the International Scientific Association for Probiotics and Prebiotics, probiotics are defined as live microorganisms that, when administered in adequate amounts confer a health benefit upon the host.; However, the magnitude of benefit depends on strain specificity, dosage, and viability [9]. Common genera include *Bifidobacterium* and *Lactobacillus*, which are found in fermented foods and the intestine of humans. Postbiotics — comprising non-viable bioactive components derived from probiotic metabolism, including short-chain fatty acids (SCFAs), bacteriocins, and cell wall fragments — exert immunomodulatory and metabolic effects and may broaden therapeutic utility [10]. This review systematically delineates the mechanistic spectrum of six well-characterized probiotic genera/species and their corresponding postbiotic derivatives to provide insights into recent advances in therapeutic strategies for CMDs. The timing of microbial therapy administration and the host environment also influence the functional activity of these microorganisms in vivo. Collectively, the mechanisms reviewed here provide a basis for advancing cardiometabolic precision medicine and developing mechanism-informed personalized interventions.

### Introduction of the Currently Existing Mainstream Probiotics

A diverse range of bacterial species includes well-characterized probiotics. This review focuses on six major genera/species: *Akkermansia muciniphila* (*A. muciniphila*), *Bifidobacterium*, *Lactobacillus*, *Bacillus*, *Enterococcus*, and *Lactococcus lactis*. These six were selected based on the following criteria: (i) a substantial body of peer-reviewed evidence from both preclinical models and clinical studies linking them to CMD-related outcomes; (ii) representation of distinct phylogenetic lineages and metabolic capabilities, allowing coverage of complementary mechanistic pathways (mucin degradation, SCFA production, bile acid metabolism, exopolysaccharide secretion, and immunomodulation); (iii) inclusion of both genus-level taxa with broad probiotic applications (*Bifidobacterium*, *Lactobacillus*, *Bacillus*, *Enterococcus*) and a species-level model organism (*A. muciniphila*) that exemplifies the translational trajectory from discovery to clinical testing; and (iv) availability of evidence on postbiotic derivatives for at least a subset of these organisms, enabling comparative discussion of live versus inactivated preparations. Comprehensive functional profiles and therapeutic applications of these six probiotics are systematically presented in Table 1.

### Molecular Mechanisms of Probiotic Intervention in Cardiometabolic Diseases

**Regulation of gut microbiota and metabolites.** Gut microbiota imbalance can contribute to obesity and diabetes risk. Functional gastrointestinal disorders are common in patients with CMDs. Probiotics can inhibit the growth and proliferation of pathogenic microorganisms through competition for nutrients and production of antimicrobial substances, thereby aiding the restoration of microbial balance [11, 12]. For example, *Bifidobacterium* and *Lactobacillus* species promote the growth of beneficial bacteria while suppressing pathogenic overgrowth [13].

Probiotics promote energy balance and control lipid metabolism through influencing the production of microbiota metabolites like SCFAs. These compounds prevent the absorption of cholesterol from the gut and prevent atherogenesis [14]. Probiotics can also alter hepatic cholesterol metabolism through changing the profile of bile acids (BAs) [15].

Elevated levels of trimethylamine N-oxide (TMAO) are linked to the development and progression of CVDs, including AS, coronary artery disease, and heart failure. Probiotics inhibit the metabolic activity of trimethylamine (TMA)-producing gut microbes, thereby decreasing the generation of TMAO and contributing to CVD prevention [16]. Beyond the live probiotic effects described above, certain postbiotic components — such as SCFAs and inactivated bacterial preparations — may independently influence these metabolic pathways, although direct comparisons between live and postbiotic interventions in CMD contexts remain limited [10].

**Regulation of gut barrier function.** Alterations in gut microbiota can affect their interaction with the immune system and the intestinal barrier. The gut microbiota is thought to play a role in CVDs [17]. Probiotics improve the function of the intestinal barrier and inhibit the release of inflammatory mediators that cause CMDs [18]. Oral administration of *Bifidobacterium longum* (*B. longum*) NK49 and *Lactobacillus plantarum* (*L. plantarum*) NK3 was shown to improve obesity and osteoporosis in mice. These strains strengthen intestinal barrier function, modulate immune cells, and reduce TNF-α expression [19]. In terms of postbiotic contributions, *A. muciniphila*-derived extracellular vesicles and the outer membrane protein Amuc_1100 have been shown to reinforce tight junction integrity independently of live bacterial cells [20, 21], illustrating the potential of postbiotic preparations to target barrier function directly.

**Regulation of Immune and Inflammatory Systems.** According to a study, probiotics actively regulate immune cell activity, thereby inhibiting the activation of pro-inflammatory TNF-α and NF-κB signaling [19]. At the same time, probiotics can modulate the immune cell function, for example, regulating TH17 and Treg cell differentiation to reduce the inflammatory response [22]. The interplay between gut microorganisms and the immune system plays a critical role in the pathogenesis of CVDs [23]. While the majority of mechanistic evidence in this area derives from studies employing live probiotics, cell wall fragments and secreted proteins from probiotic strains may also contribute to immunomodulation, highlighting the need for studies that directly compare the immune effects of viable and inactivated preparations.

### The Role of Probiotics in Cardiometabolic Diseases

Anti-obesity and lipid metabolism regulation. Probiotics may influence obesity by regulating lipid metabolism. In a representative study, *Bifidobacterium longum* subsp. *infantis* FB3-14 (*B. longum* FB3-14) was evaluated in mice fed a high-fat diet (HFD). After an eight-week intervention, FB3-14-supplemented mice exhibited significant decreases in body weight, fat mass, and abnormal serum lipid levels, thereby restoring cholesterol homeostasis and reducing low-density lipoprotein (LDL) levels. Correspondingly, the expression of lipid metabolism-related genes, including hormone-sensitive lipase (HSL), leptin, and adiponectin, was also altered. These effects were associated with a reduction in the Firmicutes-to-Bacteroidetes ratio (F/B ratio) and an increase in health-promoting bacterial species, including *A. muciniphila* and *Bifidobacterium*, suggesting that FB3-14 may be a promising functional supplement for obesity management [24].

A probiotic mix of *B. animalis* LA804 and *L. gasseri* LA806 reduced weight gain, fat accumulation, and plasma triglyceride (TG) levels in HFD-fed mice while improving lipid metabolism and decreasing inflammatory markers. While it created small changes in the composition of gut microbiota, this did not lead to the restoration of HFD-induced dysbiosis. However, it inhibited Farnesoid X Receptor (FXR) signaling in the ileum and helped in protection of lipid metabolism [25]. Furthermore, *B. longum* 070103 fermented milk reduced fasting glucose, cholesterol, leptin, and fat levels more effectively than other probiotic formulations. *B. longum* 070103 also improved glucose tolerance, insulin resistance, and liver function by modulating gut microbes and metabolites, thereby further supporting its anti-obesity effects [26].

Within the phylum Verrucomicrobiota, *A. muciniphila* represents the sole culturable genus. In obesity research, its abundance has been shown to have an inverse correlation with the body mass index (BMI). Multiple studies, including analyses of pregnant women [27], preschool children [28], and adults [29], have consistently shown that the abundance of *A. muciniphila* is lower in obese individuals compared to those with normal weight. This pattern has also been validated in animal models [30]. Despite some contrary results [31], the overall trend indicates a reduced abundance of *A. muciniphila* in individuals with obesity or overweight.

Supplementation with *A. muciniphila* may ameliorate obesity and its associated pathological features. A 2013 investigation led by Everard *et al*. demonstrated that live *A. muciniphila* administration markedly reduced adiposity, improved insulin sensitivity, attenuated adipose tissue inflammation, and lowered metabolic endotoxemia in obese mice, whereas inactivated preparations lacked efficacy in producing these benefits [30]. Further animal studies demonstrated that *A. muciniphila* supplementation also aids in weight reduction, enhances metabolism, and strengthens intestinal barrier function [32–37].

A noteworthy finding by Plovier *et al*. was that the metabolic regulatory activity of *A. muciniphila* was enhanced following pasteurization [38, 39]. In murine models of obesity, the pasteurized bacterium demonstrated superior efficacy in mitigating weight gain, fat accretion, and insulin resistance induced by a high-fat diet[38]. Subsequently, a randomized, double-blind, placebo-controlled clinical trial confirmed that daily oral supplementation with 10^10^ colony-forming units (CFU) of either live or pasteurized *A. muciniphila* over a three-month intervention period was safe and well-tolerated in overweight or obese volunteers [39].

The trial results showed that pasteurized *A. muciniphila* significantly reduced plasma total cholesterol, body weight, adipose tissue mass, and hip circumference, while also markedly improving insulin sensitivity [40]. In exploring the specific mechanisms by which *A. muciniphila* alleviates obesity, research further revealed that the heat-treated bacteria counteract diet-induced weight gain via two primary pathways: first, by increasing whole-body energy expenditure and spontaneous physical activity; and second, by lowering the levels of perilipin 2 in adipose tissue—a protein critically involved in lipid droplet dynamics and structural integrity [40]. Additionally, pasteurized *A. muciniphila* was found to increase fecal energy excretion, which may be associated with reduced carbohydrate absorption and promoted intestinal epithelial cell turnover [40].

Experimental investigations demonstrated that exposure of 3T3-L1 cells to *A. muciniphila* cell lysates significantly reduced lipid deposition and suppressed mRNA levels of adipogenic markers. Furthermore, *A. muciniphila* promotes the production of serine protease inhibitor A3G [41] within adipocytes, which subsequently inhibits adipocyte differentiation. However, research on the molecular mechanisms underlying the anti-obesity effects of *A. muciniphila* are still in nascent phases, and additional studies are required to clarify the precise molecular pathways.

Collectively, the studies reviewed above demonstrate that probiotics and postbiotics targeting obesity operate through partially overlapping yet mechanistically distinct pathways: *Bifidobacterium* strains primarily modulate the F/B ratio and hepatic lipid metabolism genes, *Lactobacillus* strains engage BSH-mediated bile acid modification and FXR signaling, while *A. muciniphila* — particularly in pasteurized form — acts through energy expenditure enhancement and adipocyte differentiation blockade. A critical gap in the current evidence is the paucity of head-to-head comparisons among these strains and between live versus postbiotic preparations within the same study, which limits the ability to rank their relative anti-obesity efficacy.

**Nonalcoholic fatty liver disease.** Probiotics show therapeutic potential in NAFLD. A study using *L. plantarum* and *B. bifidum* demonstrated reduced hepatic lipid accumulation and improved oxidative stress. These strains also enhanced cellular immune function, activated the AMPK/Nrf2 signaling pathway, and inhibited the LPS-TLR4/NF-κB pathway. Collectively, these findings suggest that *L. plantarum* and *B. bifidum* may improve intestinal health, reduce inflammation, and normalize lipid metabolism, representing a feasible therapeutic approach for NAFLD [42].

*Lactobacillus* strains participate in a variety of metabolic pathways relevant to cholesterol reduction, attenuation of inflammation, and neutralization of reactive oxygen species (ROS). *Lactobacillus* catalyze the hydrolysis of conjugated BA via bile salt hydrolase (BSH). This mechanism contributes to the formation of insoluble complexes composed of bile salts and cholesterol, which are subsequently eliminated via feces [43–45]. Moreover, *Lactobacillus* inhibits FXR signaling to enhance the expression of CYP7A1. This process transforms cholesterol to BA while preventing the reabsorption of BA [46, 47]. Furthermore, *Lactobacillus* enhances the production of SCFAs, which activates liver X receptor alpha (LXRα) to aid in regulating lipid and cholesterol metabolism in the liver [48]. On the contrary, TMAO also impedes BA synthesis by interfering with certain enzymes as a result of FXR activation, leading to the promotion of aortic lesions [49].

The evidence for probiotics in NAFLD converges on the gut-liver axis as a central regulatory node, with BSH activity and SCFA production representing the most consistently reported mechanisms across *Lactobacillus* and *Bifidobacterium* strains. However, the majority of studies have been conducted in HFD rodent models with relatively short intervention periods (8-12 weeks), and translation to human NAFLD — where disease progression occurs over years — requires validation in longer-term clinical trials. Notably absent from the current literature are studies systematically comparing single-strain versus multi-strain probiotic formulations for NAFLD outcomes.

**Type 2 diabetes mellitus.** Studies indicate notable changes in the intestinal microbiome of individuals with type 2 diabetes mellitus (T2DM), characterized by lower levels of *A. muciniphila* documented in T2DM patients relative to healthy controls [50]. As a potential therapeutic strategy, A. muciniphila supplementation has attracted attention for its ability to improve several physiological indicators linked to T2DM. In HFD-induced T2DM mouse models, supplementation with *A. muciniphila* has been reported to attenuate hyperglycemia, improve glucose tolerance, rebalance the gut microbial community, increase SCFAs production, and reinforce intestinal barrier function [30, 51]. Taken together, these findings offer solid experimental support for considering *A. muciniphila* as a candidate adjunct in diabetes management. Notably, recent evidence suggests that the metabolic benefits of *A. muciniphila* are not uniform across individuals, but instead depend strongly on its baseline abundance in the gut. In particular, patients starting with lower *A. muciniphila* levels tended to show clearer improvements after supplementation—better glycemic control, higher insulin sensitivity, and more favorable weight-related outcomes—whereas those with relatively high baseline levels showed little or no measurable benefit. These results suggest that *A. muciniphila* supplementation may be more beneficial for patient populations with lower abundances of this bacterium in their gut [52].

*A. muciniphila* appears to improve metabolic homeostasis through several, partly convergent routes. It not only reduces circulating free fatty acid levels by counteracting adiposity, but also reshapes the gut microbial community in a way that favors SCFA biosynthesis. In parallel, *A. muciniphila* can engage intestinal G-protein-coupled receptors (GPR41/43), which promotes glucagon-like peptide-1 (GLP-1) release[53, 54]. Adding to this, the bacterium secretes a GLP-1-inducing protein, P9, that activates downstream signaling cascades and further augments GLP-1 secretion [55]. Once released, GLP-1 enters the circulation and reaches the pancreas, where it binds its cognate receptor and stimulates insulin secretion; consequently, blood glucose levels decline. Beyond incretin-related effects, *A. muciniphila* also helps maintain intestinal barrier integrity by activating the ALPK1/TIFA pathway in an ADP-heptose-dependent manner. This response upregulates gene programs involved in barrier maintenance while lowering intestinal permeability, thereby limiting lipopolysaccharide (LPS)-driven chronic low-grade inflammation and the associated insulin resistance [56]. Consistently, enhanced expression of tight-junction proteins, including ZO-1, occludin, and claudin, has also been reported following *A. muciniphila* exposure[57]. Notably, specific *A. muciniphila*-derived factors—such as the outer membrane protein Amuc_1100 [58] and extracellular vesicles [33]—have been implicated in reinforcing gut barrier structure. From this perspective, *A. muciniphila* and its bioactive components represent a plausible therapeutic avenue for T2DM, operating through a combination of increased insulin secretion and reduced insulin resistance.

**Cardiovascular diseases (Atherosclerosis and Hypertension).** The administration of *Lactobacillus* has been shown to modify the composition of gut microbiota by lowering the F/B ratio and substantially increasing the abundance of Bacteroides, *Lactobacillus*, and *Bifidobacterium*. *Lactobacillus* participates in metabolic processes involving the host’s digestive repertoire. The gut microbes generate TMA, SCFAs, LPS, BA, and other metabolites that play important roles in CVD development [59].

*Lactobacillus* strains can suppress the hepatic conversion of TMA to TMAO, which matters because TMAO is widely regarded as a cardiovascular risk factor [16, 60]. Recent work further indicates that *L. plantarum* ZDY04 markedly lowers both serum TMAO and cecal TMA in mice. Mechanistically, this effect appears to involve remodeling of the gut microbial community—particularly by shifting the abundance of taxa such as *Lachnospiraceae* and *Erysipelotrichaceae*—which is accompanied by an attenuation of AS. Along similar lines, a recent clinical study reported that supplementation with *L. plantarum* GLP3 reduced circulating TMAO levels in patients at very high cardiovascular risk [61].

Supplementation with *Lactobacillus* tends to enrich gut microbes associated with SCFA production, including *Roseburia*, *Ruminococcus*, and *Eubacterium*, thereby supporting greater SCFA generation through dietary fiber fermentation [62,63]. SCFAs, in turn, play an important role in sustaining cardiovascular health. LPS, a major constituent of the outer membrane of Gram-negative bacteria, is also implicated in the pathogenesis of HTN, obesity, and T2DM. In this context, *Lactobacillus* has been reported to lower circulating LPS levels [64, 65]. In addition, disturbances in BA metabolism have been linked to dyslipidemia, CVDs, and diabetes. *Lactobacillus* appears to influence BA biotransformation by enhancing microbial BSH activity, which promotes the generation of primary free BAs and facilitates the formation of microbially derived secondary BAs and related intermediates [66, 67]. Moreover, *Lactobacillus* can strengthen intestinal barrier function, in part via antimicrobial effects, while also shaping gut microbiota composition and modulating intestinal permeability. Reported changes include an increase in Bacteroidetes, a reduced relative abundance of Actinobacteria, and preservation of barrier integrity through protection of key tight-junction proteins such as claudin-1, occludin, and ZO-1, collectively contributing to beneficial outcomes [68–70]. Along related lines, Lee *et al*. engineered a recombinant *Lactococcus lactis* strain to express the Ling-Zhi immunomodulatory protein LZ8. This expressed protein showed anti-inflammatory activity, associated with reduced protein levels and gene expression linked to lipid metabolism and inflammatory pathways in the aorta. Notably, administration of this strain ameliorated AS and NAFLD in rabbits fed a HFD [71, 72].

Recent studies have shown that *Bacillus amyloliquefaciens* exerts protective effects against AS by promoting anti-inflammatory M2 macrophage polarization, reducing foam cell formation, and alleviating hypercholesterolemia, hyperglycemia, hyperlipidemia, and inflammation in ApoE-/- AS mice [73]. Previous studies have demonstrated the therapeutic potential of exopolysaccharides (EPS) secreted by *B. subtilis* sp. in diabetes-associated CVDs. The findings demonstrated that EPS notably enhanced the regulation of blood glucose levels and rectified lipid metabolism aberrations in streptozotocin (STZ)-induced diabetic rats. Specifically, it led to a reduction in blood glucose concentrations, total cholesterol levels, LDL levels, and TG amounts. Simultaneously, it significantly increased the levels of high-density lipoprotein (HDL) [74]. Follow-up clinical studies have shown that supplementation with *B. subtilis* DE111 may improve blood lipid profiles and endothelial function in healthy adults [75]. Furthermore, *B. subtilis* serves a vital function in the fermentation process of soybeans, yielding natto—a functional food with significant health benefits. Natto not only inhibits AS and thrombus formation but also effectively lowers the occurrence of CVDs by reducing body fat accumulation and lowering the risk of hyperlipidemia [76, 77].

A growing body of evidence links *A. muciniphila* with AS. In ApoE-/- mouse models, *A. muciniphila* has been reported to slow atherosclerotic plaque progression, with accompanying improvements in cardiac function and reductions in body weight. It also appears to reshape systemic inflammation, lowering circulating IL-6 while increasing IL-10. Interestingly, *A. muciniphila* supplementation has been associated with higher abundances of *Lactobacillaceae* in the gut microbial community [78, 79]. Additional work suggests that *A. muciniphila* administration enhances resistance to cold-induced atrial fibrillation (AF), potentially by suppressing the synthesis of TMA and its oxidized product TMAO [80]. Consistently, *A. muciniphila* abundance is reported to be inversely correlated with blood pressure (BP) [39, 81]. In related probiotic studies, *Bifidobacterium lactis* M8 and *Lactobacillus rhamnosus* M9 reduced BP and shifted metabolite profiles in high-fructose-fed mice [82]. The *L. rhamnosus* GG strain has likewise been shown to mitigate HTN progression by modulating TMAO levels and CD4^+^ T cell-driven type I inflammatory responses [60]. At present, the physiological activity of *A. muciniphila* in CMDs has been partially validated. Using the spontaneously hypertensive rat (SHR) model, investigators further found that milk fermented with *Lactococcus lactis* NRRL B-50571 and NRRL B-50572 lowered plasma LDL cholesterol and TG levels, suggesting a favorable impact on cardiovascular health [83]. A systematic summary of animal studies of probiotic interventions for CMDs is provided in Table 2, and corresponding clinical studies are presented in Table 3. These tables reveal several overarching patterns: (i) *Lactobacillus* and *Bifidobacterium* strains are the most extensively studied probiotics in CVD contexts, with consistent reductions in TMAO, LDL-cholesterol, and inflammatory markers; (ii) *A. muciniphila* stands out as the only species for which pasteurized (postbiotic) preparations have been directly compared with live forms in both animal models and clinical trials, with the pasteurized form showing equal or superior efficacy; (iii) the majority of clinical studies remain small (n < 60) and of short duration (≤12 weeks), limiting statistical power and the ability to detect effects on hard cardiovascular endpoints; and (iv) inter-study variability in probiotic dose, formulation, and concomitant dietary background makes cross-study comparisons challenging and underscores the need for standardization in future trials.

**Integrated perspective: Probiotics in CMD multimorbidity management.** CMDs seldom occur in isolation but rather as a cluster that involves metabolic and cardiovascular pathologies that overlap. For instance, the dysfunction in heart failure with preserved ejection fraction (HFpEF) often triggers due to NAFLD and T2DM. Probiotics modulate systemic chronic low-grade inflammation via “multi-target” effect Targeting the LPS-TLR4 signaling pathway via microbial interventions could improve insulin sensitivity and reduce lipotoxicity in the liver and heart. This provides unified treatment approach for patients with multiple cardiometabolic comorbidities.

Future clinical applications should transition from organ-specific interventions to systemic modulation of the “Gut-Heart-Liver Axis”. In particular, it remains essential to clarify how defined probiotic strains (for instance, *A. muciniphila*) can simultaneously shape glucose disposal in skeletal muscle, hepatic lipid oxidation, and endothelial function within the vasculature. From this perspective, such an integrative framework fits well with the central aims of CMD management, because it targets the shared pathophysiological underpinnings that connect these closely intertwined disorders.

### Synergy between Probiotics and Next-Generation Pharmacotherapies

**SGLT2 inhibitors and the SCFA connection.** Recent evidence indicates that Sodium-glucose cotransporter 2 inhibitor (SGLT2i) like empagliflozin may modulate the gut microbiota by increasing the abundance of SCFA producers. Use of probiotics (such as *Bifidobacterium* or *F. prausnitzii*) might enhance this effect, leading to increased systemic anti-inflammatory effects resulting in better protection against heart failure with HFpEF [84, 85]. The cooperation may be a “double-hit” operation on the gut-heart-liver axis [86]. However, it should be noted that current evidence for these synergistic effects derives primarily from animal models and correlative microbiome analyses; dedicated randomized controlled trials designed to test probiotic-SGLT2i combinations are not yet available, and the clinical magnitude of any additive benefit remains to be quantified.

**GLP-1 Receptor agonists and the *Akkermansia* “Double Hit”.** GLP-1 receptor agonists (GLP-1RA; *e.g.*, semaglutide) activate GLP-1 signaling pharmacologically, whereas *A. muciniphila* can promote endogenous GLP-1 release through its secreted protein P9 [55]. By targeting exogenous receptor activation and endogenous production, the combination of these two may lead to weight loss and more stable glycemic control. Probiotics may prevent common GI adverse effects like nausea and bloating due to GLP-1RA therapy through restoration of intestinal barrier function and effect on motility [86-88].

**Statins and BSH-active probiotics.** To manage lipids, combining statins with the BSH-active *Lactobacillus* strains may lead to synergistic improvement in LDL-C levels by inhibiting hepatic cholesterol synthesis and improving intestinal BA/cholesterol excretion [89]. A decrease in statin dose may lessen the risk for statin-associated muscle symptoms [90].

### Timing and Environmental Influences on the Use of Probiotics

Probiotics have clear promise for reshaping the gut microbiota and, by extension, supporting host health; however, their benefits depend strongly on when they are introduced and the context in which they are used. To begin with, timing matters. Probiotic administration early in life may help guide the assembly of a resilient microbial community, which is particularly relevant for preterm infants and neonates. In this period, strains such as *Bifidobacterium longum* subsp. *infantis* and *Lactobacillus acidophilus* have been reported to reduce colonization by multidrug-resistant bacteria and to strengthen immune function [91, 92]. Likewise, when antibiotics are required, concurrent probiotic supplementation can lessen microbiota disruption and reduce complications such as *Clostridioides difficile*-associated diarrhea [93]. In older adults, certain probiotics (including *Bifidobacterium* spp.) may improve intestinal barrier integrity and immune responsiveness, which could, at least in principle, help slow the trajectory of age-related pathology[94].

Environmental determinants also play a major role in shaping probiotic performance, with diet, pollutant exposure, and lifestyle behaviors among the most influential. Dietary patterns are a straightforward example: high-fiber, low-fat diets generally support probiotic colonization, whereas fat- and sugar-rich diets tend to promote dysbiosis and may blunt probiotic effects [95]. Exposure to environmental contaminants, such as heavy metals, can impair intestinal barrier function; probiotics may partially offset these harms by competing with pathogens and reinforcing barrier integrity [96]. Finally, lifestyle factors should not be overlooked. Chronic stress and sleep deprivation often worsen microbial imbalance, while moderate physical activity is associated with greater microbial diversity and a more stable ecosystem [97].

### Conclusions and Future Perspectives

**Fig. 1:**
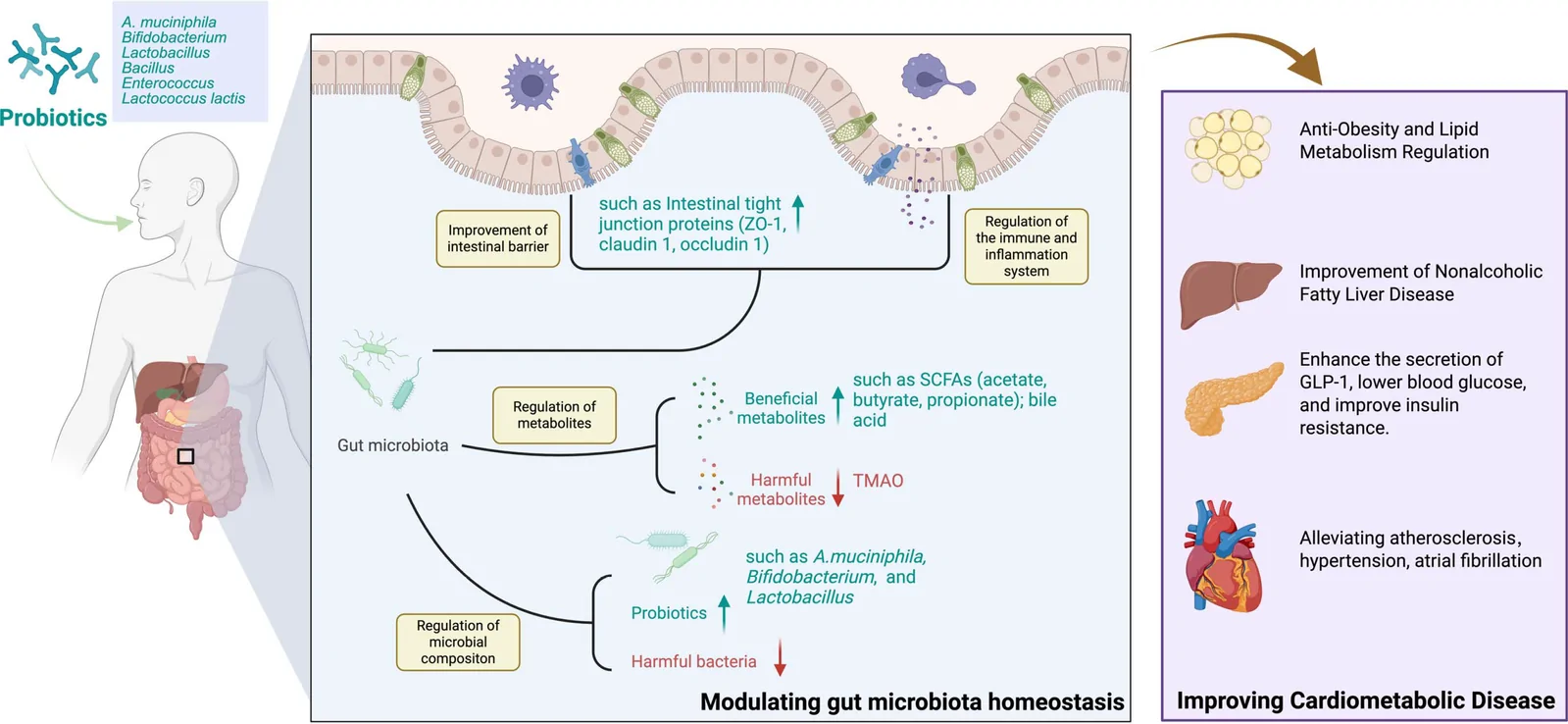
Mechanistic pathways of probiotics in CMDs via gut microbiota modulation. Probiotics directly counteract CMDs by restoring microbial balance, enhancing the abundance of beneficial bacteria and decreasing harmful species. Furthermore, probiotics mediate therapeutic effects through enhancing the biosynthesis of SCFAs and BAs while inhibiting TMAO formation. Additionally, modulation of the immune-inflammatory system and intestinal barrier function synergistically contribute to CMD pathophysiology.

Current evidence indicates that probiotics can contribute to the management of CMDs through modulation of microbiome-host crosstalk, influencing SCFA metabolism, BA signaling, and immune homeostatic balance. Their health benefits are particularly evident in obesity, metabolic syndrome, and NAFLD (Fig. 1). Postbiotic preparations — most notably pasteurized *A. muciniphila* — have emerged as a complementary strategy that may offer advantages in terms of safety, standardization, and, in some cases, enhanced efficacy relative to live preparations. However, several important challenges remain.

First, probiotic effects are strain-specific; therefore, appropriate strain selection is critical for clinical efficacy. The majority of commercially available probiotic products are not supported by rigorous strain-specific evidence, and the functional mechanisms of different strains remain incompletely characterized. Second, many clinical trials to date have been restricted to small sample sizes (typically fewer than 100 participants), limiting their statistical power and generalizability. Third, the composition and function of the gut microbiota are highly individualized, which contributes to inter-individual variability in probiotic responsiveness. This variability underscores the need for personalized treatment strategies that incorporate host genetic factors, dietary patterns, and baseline gut microbiota characteristics.

In the emerging paradigm of precision cardiometabolic medicine, microbial strains should be selected based on scientific evidence rather than empirical application. As illustrated in Table 4, we propose an illustrative, hypothesis-generating framework in which study-specific metabolic phenotypes may guide the selection of candidate probiotic or postbiotic interventions for future testing. We do not propose universal numerical cutoffs for TMAO or *A. muciniphila* abundance, because currently available evidence has not validated such thresholds for clinical decision-making; reported associations and abundance ranges differ markedly across cohorts and sequencing pipelines. The doses listed in Table 4 are representative values from the cited studies rather than recommended clinical regimens, and preclinical (mouse) doses are not directly translatable to humans. The cited studies support associations, mechanistic hypotheses, or preliminary feasibility rather than treatment-selection rules, and measurements of TMAO, LPS, SCFAs, bile acids, and microbial relative abundance are affected by assay platform, fasting and dietary status, renal function, sample handling, and analytical pipeline. Prospective studies with prespecified assays, doses, and clinically relevant outcomes are required before phenotype-guided microbial interventions can be considered in practice.

Future research should prioritize the following directions: (i) employing multi-omics technologies (metagenomics, metabolomics, and proteomics) to elucidate the precise molecular mechanisms of key functional strains and their postbiotic products; (ii) investigating the synergistic effects of probiotics combined with prebiotics, pharmacotherapies, or other biological agents in well-designed factorial trials; (iii) leveraging gene-editing technologies to enhance the functionality and safety of probiotic strains; (iv) conducting large-scale, multi-center clinical trials with robust long-term safety monitoring; and (v) developing artificial intelligence (AI)-driven predictive models that integrate microbiome, host phenotypic, and metabolic data to enable precise, personalized interventions.

## Figures and Tables

**Table 1:** Introduction of the currently existing mainstream probiotics.

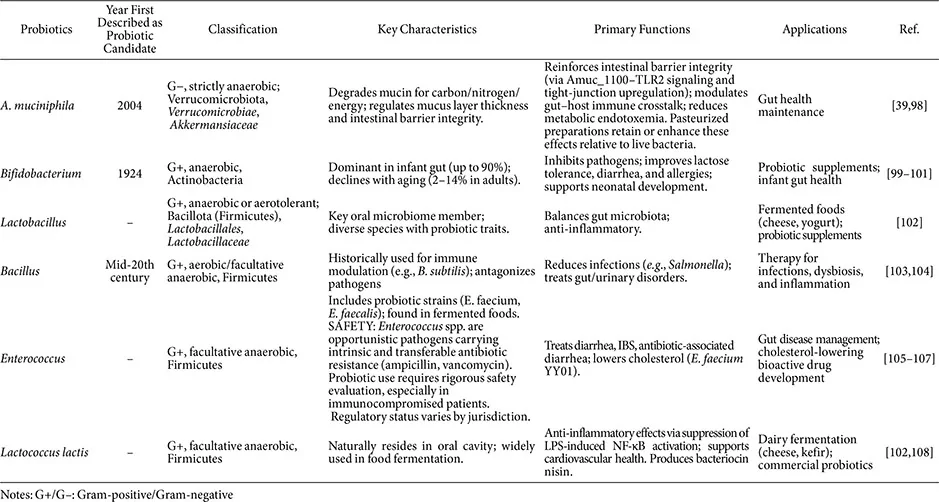

**Table 2:** Experimental investigations of probiotics and postbiotics on CMDs.

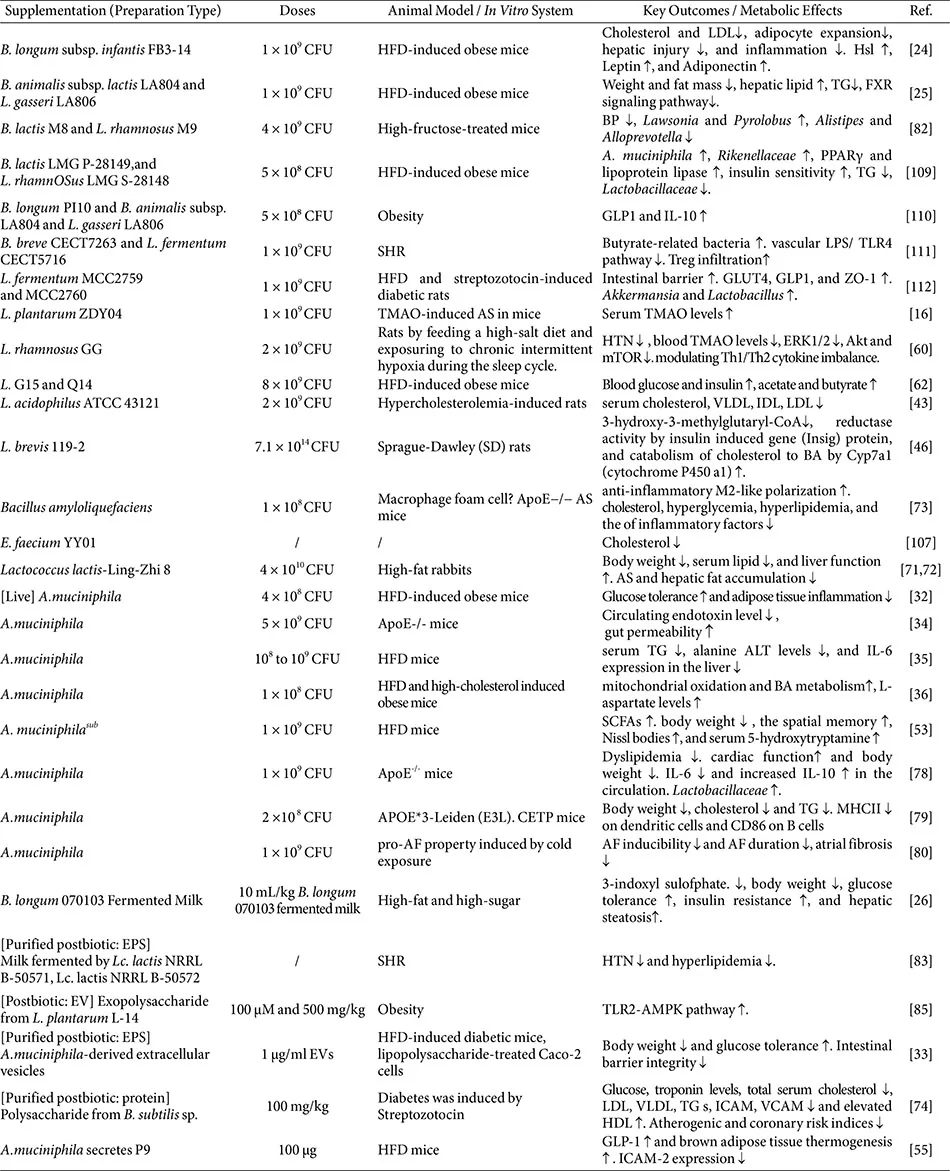

**Table 3:** Clinical effects of probiotics and postbiotics on CMDs.

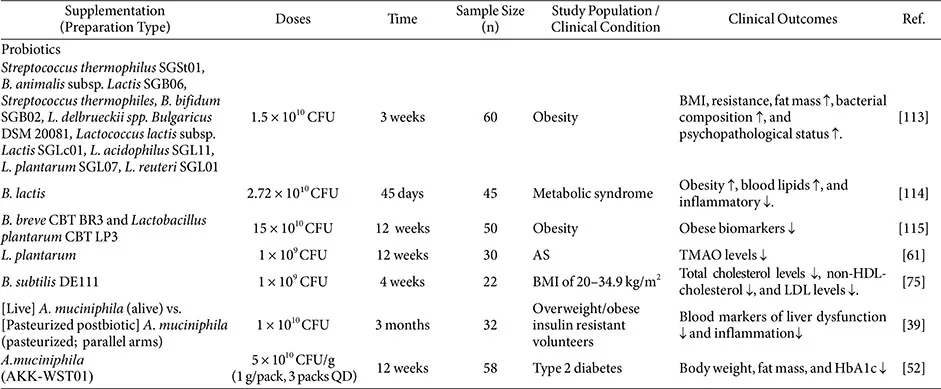

**Table 4:** Proposed Hypothesis-Generating Framework for Precision Probiotic and Postbiotic Interventions in Cardiometabolic Disease Based on Baseline Metabolic Phenotypes.